# ﻿Morphology and multigene phylogeny reveal three new species of *Samsoniella* (Cordycipitaceae, Hypocreales) from spiders in China

**DOI:** 10.3897/mycokeys.101.111882

**Published:** 2024-02-02

**Authors:** Ting Wang, Jun Li, Xiaoyun Chang, Zengzhi Li, Nigel L. Hywel-Jones, Bo Huang, Mingjun Chen

**Affiliations:** 1 Anhui Provincial Key Laboratory for Microbial Pest Control, Anhui Agricultural University, Hefei 230036, China Anhui Agricultural University Hefei China; 2 Natural Resources and Planning Bureau of Bengbu City, Bengbu, Anhui 233000, China Natural Resources and Planning Bureau of Bengbu City Bengbu China; 3 Zhejiang BioAsia Institute of Life Sciences, 1938 Xinqun Road, Economic and Technological Development Zone, Pinghu, Zhejiang 314200, China Zhejiang BioAsia Institute of Life Sciences Pinghu China

**Keywords:** Araneogenous fungi, *Isaria*-like, *Samsoniella*, taxonomy

## Abstract

The genus *Samsoniella* was erected based on orange cylindrical to clavate stromata, superficial perithecia and conidiophores with *Isaria*-like phialides and to segregate them from the *Akanthomyces* group. In this study, based on morphological features and multigene (SSU, LSU, *TEF*, *RPB1* and *RPB2*) phylogenetic analysis six *Samsoniella* species parasitizing spiders were collected in China. Three of them belong to known species *S.alpina*, *S.erucae* and *S.hepiali*. Three new species *S.anhuiensis***sp. nov.**, *S.aranea***sp. nov.** and *S.fusiformispora***sp. nov.** are illustrated and described. They are clearly distinct from other species in *Samsoniella* occurring in independent subclades. Furthermore, among the four insect-pathogenic fungi specimens collected from similar sites, three of them were identified as the new species described below. Our study significantly broadens the host range of *Samsoniella* from Insecta to Arachnida, marking a noteworthy expansion in understanding the ecological associations of these fungi. Additionally, the identification of both mononematous and synnematous conidiophores in our study not only expands the knowledge of *Samsoniella* species but also provides a basis for future research by comparing the ecological significance between these conidiophore types. In conclusion, our study enhances the understanding of *Samsoniella* diversity, presenting a refined phylogenetic framework and shedding light on the ecological roles of these fungi in spider parasitism.

## ﻿Introduction

The genus *Isaria* Pers. was established by Persoon (1794) with *I.farinosa* (Pers.) Fr. as the type species (Hodge et al. 2005). *Isaria* is characterized by the formation of branched synnemata that give rise to flask-shaped phialides produced in whorls. For a considerable period, *Isaria* has been considered the asexual morph of *Cordycepssensu stricto*, a classification within the family Cordycipitaceae, which encompasses numerous species featuring pallid or brightly pigmented, fleshy stromata (Sung et al. 2007; Maharachchikumbura et al. 2015). Samson (1974) transferred some species including *I.farinosa* to *Paecilomyces*Bainer (1907). However, Hodge et al. (2005), based on morphological and molecular phylogenetic studies, moved *Paecilomycesfarinosa* back to *Isaria* re-establishing the type as *Isariafarinosa* (Holmsk.) Fr. Most of the insect-pathogenic mesophilic Paecilomyces species in sect. Isarioidea of Samson (1974) were transferred to *Isaria* (Luangsa-ard et al. 2004, 2005; Gams et al. 2005). Nonetheless, Kepler et al. (2017) proposed the rejection of the genus *Isaria* due to the polyphyletic distribution of *Isaria* species. Recently, molecular phylogenetic analysis, has shown that some *Isaria*-like fungi are distributed in the genus *Akanthomyces* of the family Cordycipitaceae, forming monophyletic branches and are closely related to the genus *Akanthomyces*. Mongkolsamrit et al. (2018) established this phylogenetic branch as a new genus *Samsoniella* Mongkols., Noisrip., Thanakitp., Spatafora & Luangsa-ard. They accommodated three species of Lepidoptera entomopathogenic fungi in the genus; *S.alboaurantia* (G. Sm.) Mongkolsamrit, *S.aurantia* Mongkolsamrit and *S.inthanonensis* Mongkolsamrit. The three species have orange cylindrical to clavate stromata, superficial perithecia and orange conidiophores with *Isaria*-like phialides and hyaline conidia.

Over the past seven years, there has been extensive research on the species diversity within the genus *Samsoniella*, possibly driven by the significant medical and ecological value associated with certain species in the genus. In a follow-up study, Wang et al. (2020a) documented nine new species within the genus *Samsoniella*. Specifically, *Paecilomyceshepiali* Chen, formerly misconstrued as the asexual counterpart of *Ophiocordycepssinensis*, demonstrated the ability to produce *Isaria*-like phialides. The perplexing taxonomic status of *P.hepiali* prompted taxonomists to reconsider its classification. Wang et al. (2020a) determined that the most suitable systematic position for *P.hepiali* is within the genus *Samsoniella*. Consequently, they proposed the new taxonomic combination *S.hepiali* for this species. Subsequently, Chen et al. (2020) described three additional species of *Samsoniella*. Furthermore, phylogenetic analysis led to the repositioning of strains previously identified as *I.farinosa*. Notably, strains CBS 240.32 and CBS 262.58 were integrated into the genus *Samsoniella* and redesignated as *S.alboaurantia* (Mongkolsamrit et al. 2018; Chen et al. 2021). Similarly, strains OSC 111005 and OSC 111006 were reassigned to *S.farinosa* Wang (Wang et al. 2020b). More recently, Chen et al. (2021, 2022, 2023), Wang et al. (2022), Wang et al. (2023) and Crous et al. (2023) contributed descriptions of fifteen additional novel *Samsoniella* species. Consequently, the genus *Samsoniella* now comprises a total of thirty-one recognized species.

We carried out a series of surveys for spider pathogenic fungi in China. A total of seven spider cadavers infected by *Samsoniella* were collected and isolated. Based on morphological and molecular phylogenetic analyses, three were identified as *S.alpina*, *S.erucae*, and *S.hepiali*. However, the other four strains represented four new species, which are described here as *S.anhuiensis* sp. nov., *S.aranea* sp. nov. and *S.fusiformispora* sp. nov. Among the four insect-pathogenic fungi specimens collected from the same sites, three of them were identified as the new species described below. Our study enhances the understanding of *Samsoniella* diversity, presenting a refined phylogenetic framework and shedding light on the ecological roles of these fungi in spider parasitism.

## ﻿Materials and methods

### ﻿Sample collection, isolation and morphological observations

The majority of spider specimens infected by fungi were collected from all over China. Four specimens were collected from the Jingting Mountains National Forest Park, Anhui Province, southeastern China. Four specimens were collected from the Jinggang Mountains National Nature Reserve, Jiangxi Province, southeastern China. One specimen was collected from the Maiji National Forest Park, Gansu Province, northwestern China. One specimen was collected from the Yaoluoping National Forest Park, Anhui Province, southeastern China, and one specimen was collected from the Wanfo Mountains, Anhui Province, southeastern China. Several insect specimens infected by fungi were collected from sites similar to those where spider specimens were collected. The collections were noted and photographed in the field, then carefully deposited in plastic boxes and returned to the laboratory. Fungal cultures were isolated from fresh conidia or mycelia from spider cadavers. Pure cultures were established and incubated on fresh potato dextrose agar (PDA) plates and grown at 25 °C for 2 weeks. The fresh structures of specimens and isolated strains were mounted in water for measurements and lactophenol cotton blue solution for microphotography following Wang et al. (2020a). Features such as size and shape of conidia, colony color in culture, were made from squash mounts and sections made from fresh specimen and culture grown on oatmeal agar (OA, Difco), PDA and one quarter strength SDAY (SDAY/4, Difco) (Bischoff et al. 2009). The color of the cultures was characterized using the Naturalist’s Color Guide (Smith 1975). Microscopic observations were made from squash mounts and sections made from fresh material using a ZEISS Axiolab 5 microscope. All samples and strains studied here were deposited in the Research Center for Entomogenous Fungi (RCEF) of Anhui Agricultural University.

### ﻿DNA extraction, PCR amplification and sequencing

Total genomic DNA was extracted from cultured mycelia with CTAB method (Liu et al. 2001), then stored in -20 °C. Two gene regions, namely the small subunit ribosomal RNA (SSU) and large subunit ribosomal RNA (LSU) were sequenced from the cell nuclei, and three protein coding genes, translation elongation factor-1a (TEF) and the largest and second largest subunits of RNA polymerase II (*RPB1* and *RPB2*) were used in this study. The SSU and LSU were amplified with NS1/NS4 (White et al. 1990) and LROR (Vilgalys and Hester 1990)/LR7(Hopple 1994). The *TEF* with 983F/2218R (Rehner and Buckley 2005), *RPB1* with CRPB1/RPB1–Cr (Castlebury et al. 2004) and *RPB2* with fRPB2–7CR /fRPB2–5F (Liu et al. 1999) were amplified. PCR reactions of the five nuclear loci were carried out in 25 μL reaction mixture containing 12.5 μL 2× Taq Plus MasterMix (CoWin Biosciences, Beijing, China), 1 μL of each primer (10 μM), 1.5 μL of template DNA (1–2 ng) and 9 μL of sterile water. PCR cycle conditions were as previously described (Sung et al. 2007). PCR products were purified and sequenced by Sangon Company (Shanghai, China). The resulting sequences were checked manually, then submitted to GenBank.

### ﻿Sequence alignment and phylogenetic analyses

The sequences in this study were uploaded to BLAST and searched in the GenBank database to determine probable taxa. DNA sequences generated in this study were assembled and edited using version 6.0. DNASTAR. Generated SSU, LSU, *TEF*, *RPB1* and *RPB2* sequences were aligned with those published by Chen et al. (2020) and Wang et al. (2020a) and others downloaded from GenBank were used as a dataset of taxa in *Samsoniella* and closely related *Samsoniella* groups (Table 1). Sequences of the genus *Akanthomyces* (*A.aculeatus* HUA772 and HUA 186145) were chosen as the outgroup. Multiple sequence alignment was conducted with MAFFT 7.3.13 (Katoh and Standley 2013). The final sequence alignment of the combined dataset was used for analyses using Maximum Likelihood (ML) and Bayesian Inference (BI) to infer their phylogenetic relationships.

**Table 1.** Species, strain numbers, accession numbers and origins of *Samsoniella* and related taxa used in this study, new sequences were shown in bold.

Species	Strain No.	GenBank accession No.				
		SSU	LSU	*TEF*	*RPB1*	*RPB2*
*Akanthomycesaculeatus*	HUA772	KC519368	KC519370	–	–	–
*A.aculeatus*	HUA186145^T^	MF416572	MF416520	MF416465	–	–
A.cf.coccidioperitheciatus	NHJ 5112	EU369109	EU369043	EU369026	EU369066	–
*A.coccidioperitheciatus*	NHJ 6709	EU369110	EU369042	EU369025	EU369067	EU369086
*A.farinosa*	CBS541.81	MF416606	MF416553	–	MF416655	–
*A.lecanii*	CBS101247	AF339604	AF339555	DQ522359	DQ522407	DQ522466
*A.muscarius*	CBS 143.62	KM283774	KM283798	KM283821	KM283841	KM283863
*Beauveriabassiana*	ARSEF1564^T^	–	–	HQ880974	HQ880833	HQ880905
*B.brongniartii*	ARSEF 617^T^	–	–	HQ880991	HQ880854	HQ880926
	BCC 16585	–	JF415967	JF416009	JN049885	JF415991
*B.staphylinidicola*	ARSEF 5718	EF468981	EF468836	EF468776	EF468881	–
*Cordycepsfarinosa*	CBS111113	AY526474	MF416554	GQ250022	MF416656	GU979973
*C.militaris*	OSC 93623	AY184977	AY184966	DQ522332	DQ522377	AY545732
*Isaria* sp.	spat 09-050	MF416613	MF416559	MF416506	MF416663	MF416457
	spat 09-051	MF416614	MF416560	MF416507	MF416664	MF416458
*Samsoniellaalboaurantium*	CBS 240.32	JF415958	JF415979	JF416019	JN049895	JF415999
	CBS 262.58	–	–	MF416497	MF416654	MF416448
*S.alpina*	YFCC 5818	MN576753	MN576809	MN576979	MN576869	MN576923
	YFCC 5831	MN576754	MN576810	MN576980	MN576870	MN576924
***S.alpina***	**RCEF0643**	–	–	**OM482385**	–	–
***S.anhuiensis***	**RCEF2830**	**OM268843**	**OM268848**	**OM483864**	**OM751889**	–
	**RCEF2590**	**OR978313**	**OR978316**	**OR966516**	**OR989964**	–
*S.antleroides*	YFCC 6016	MN576747	MN576803	MN576973	MN576863	MN576917
	YFCC 6113	MN576748	MN576804	MN576974	MN576864	MN576918
***S.aranea***	**RCEF2831**	**OM268844**	**OM268849**	**OM483865**	**OM751882**	**OM802500**
	**RCEF2868**	**OM268845**	**OM268850**	**OM483866**	**OM751883**	**OM802501**
	**RCEF2870**	**OR978314**	**OR978317**	**OR966517**	**OR989965**	**OR989966**
*S.aurantia*	TBRC 7271^T^	–	MF140728	MF140846	MF140791	MF140818
	TBRC 7273	–	–	MF140844	–	MF140816
*S.cardinalis*	YFCC5830	MN576732	MN576788	MN576958	MN576848	MN576902
	YFCC 6144	MN576730	MN576786	MN576956	MN576846	MN576900
*S.cristata*	YFCC6021	MN576735	MN576791	MN576961	MN576851	MN576905
	YFCC6023	MN576736	MN576792	MN576962	MN576852	MN576906
*S.coccinellidicola*	YFCC8772	ON563166	ON621670	ON676514	ON676502	ON568685
	YFCC8773	ON563167	ON621671	ON676515	ON676503	ON568686
*S.coleopterorum*	A19502	–	–	MT642602	MT642603	MN101587
*S.duyunensis*	DY09162	–	OQ363114	OQ398146	–	–
	DY07501	–	OR263307	OR282780	OR282773	OR282776
	DY07502	–	OR263427	OR282781	–	OR282777
*S.erucae*	KY11121	–	ON502835	ON525425	–	ON525424
	KY11122	–	ON502822	ON525427	–	ON525426
***S.erucae***	**RCEF2595**	**OM268842**	**OM268847**	**OM483863**	**OM751888**	–
	**RCEF2592**	–	–	**OR966518**	–	–
*S.farinosa*	OSC111005	DQ522558	DQ518773	DQ522348	DQ522394	–
	OSC111006	EF469127	EF469080	EF469065	EF469094	–
*S.farinospora*	YFCC8774	ON563168	ON621672	ON676516	ON676504	ON568687
	YFCC9051	ON563169	ON621673	ON676517	ON676505	ON568688
***S.fusiformispora***	**RCEF5406**	**OM268846**	**OM268851**	**OM483867**	**OM751890**	–
	**RCEF2588**	**OR978312**	**OR978315**	**OR966515**	–	–
*S.guizhouensis*	KY11161	–	ON502830	ON525429	–	ON525428
	KY11162	–	ON502846	ON525431	–	ON525430
*S.haniana*	YFCC8769	ON563170	ON621674	ON676518	ON676506	ON568689
	YFCC8770	ON563171	ON621675	ON676519	ON676507	ON568690
	YFCC8771	ON563172	ON621676	ON676520	ON676508	ON568691
*S.hepiali*	YFCC 5823	MN576745	MN576801	MN576971	MN576861	MN576915
	YFCC 5828	MN576744	MN576800	MN576970	MN576860	MN576914
***S.hepiali***	**RCEF1481**	**OL854202**	–	**OM482386**	–	–
*S.hymenopterorum*	A19521	–	–	MN101588	MT642603	MT642604
	A19522	–	–	MN101591	MN101589	MN101590
*S.inthanonensis*	TBRC 7915	–	MF140725	MF140849	MF140790	MF140815
*S.kunmingensis*	YHH16002	MN576746	MN576802	MN576972	MN576862	MN576916
*S.lanmaoa*	YFCC6148^T^	MN576733	MN576789	MN576959	MN576849	MN576903
	YFCC6193	MN576734	MN576790	MN576960	MN576850	MN576904
*S.lepidopterorum*	DL10071	–	–	MN101594	MN101592	MN101593
	DL10072	–	–	MT642606	–	MT642605
*S.neopupicola*	KY11321	–	ON502839	ON525433	–	ON525432
	KY11322	–	ON502833	ON525435	–	ON525434
*S.pseudogunnii*	GY407201	–	MZ827010	–	–	–
	GY407202	–	MZ831865	–	–	–
*S.pseudotortricidae*	YFCC9052	ON563173	ON621677	ON676521	ON676509	ON568692
	YFCC9053	ON563174	ON621678	ON676522	ON676510	ON568693
*S.pupicola*	DY101681	–	MZ827009	MZ855231	–	MZ855237
	DY101682	–	MZ827635	MZ855232	–	MZ855238
*S.ramosa*	YFCC6020^T^	MN576749	MN576805	MN576975	MN576865	MN576919
*S.sinensis*	YFCC8766	ON563175	ON621679	ON676523	ON676511	ON568694
	YFCC8767	ON563176	ON621680	ON676524	ON676512	ON568695
	YFCC8768	ON563177	ON621681	ON676525	ON676513	ON568696
*S.tiankengensis*	KY11741	–	ON502838	ON525437	–	ON525436
	KY11742	–	ON502841	ON525439	–	ON525438
*S.tortricidae*	YFCC6013	MN576751	MN576807	MN576977	MN576867	MN576921
	YFCC6131	MN576750	MN576806	MN576976	MN576866	MN576920
*S.vallis*	DY07241	–	OR263306	OR282778	OR282772	OR282774
	DY07242	–	OR263308	OR282779	–	OR282775
	DY091091	–	OR263428	OR282782	–	–
	DY091092	–	OR263431	OR282783	–	–
*S.winandae*	TBRC17511	–	OM491231	OM687896	OM687901	OM687899
*S.winande*	TBRC17512	–	OM491232	OM687897	OM687902	OM687900
*S.yunnanensis*	YFCC 1527	MN576756	MN576812	MN576982	MN576872	MN576926
	YFCC 1824	MN576757	MN576813	MN576983	MN576873	MN576927

Boldface: data generated in this study.

Phylogenetic inference was done according to Maximum Likelihood (ML) using RAxML version 8 (Stamatakis 2014) and Bayesian Inference (BI) using MrBayes v.3.2 (Ronquist et al. 2012). For the ML analysis, we used the GTRCAT model for all partitions, in accordance with recommendations in the RAxML manual against the use of invariant sites and 1000 rapid bootstrap replicates. The GTR+I+G model was selected by MrModeltest 2.2 (Darriba et al. 2012) as the best nucleotide substitution model for the Bayesian analysis. Four MCMC chains were executed simultaneously for 2000,000 generations, sampling every 100 generations. Finally, phylogenetic trees were visualized using the Interactive Tree of Life (iTOL) (https://itol.embl.de) online tool (Letunic and Bork 2016).

## ﻿Results

### ﻿Phylogenetic analysis

**Figure 1.**
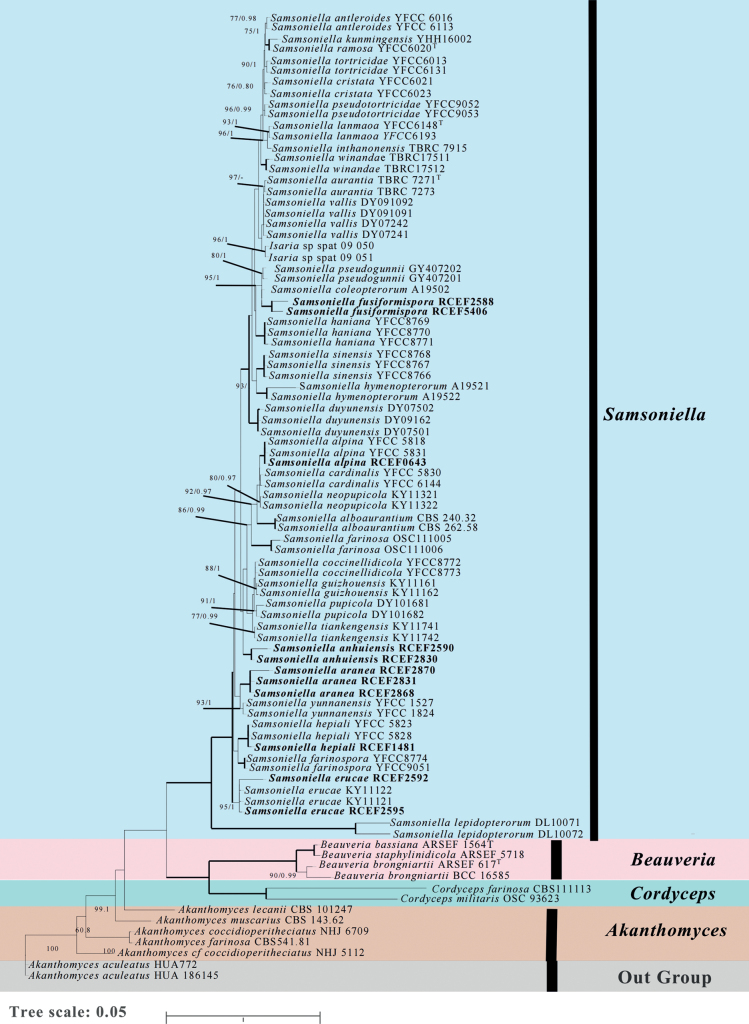
Phylogenetic relationships between the genus *Samsoniella* and closely-related species, based on multigene dataset (SSU, LSU, *TEF*, *RPB1* and *RPB2*) for maximum likelihood/ Bayesian method. Note: The ML tree presented here, and the node support rate of the two methods is displayed on the branches. The maximum likelihood support values /Bayesian posterior probabilities value (≥75%/0.75) are shown, and bold lines mean support for the two analyses were 98%. The typical strain of the species is marked with the superscript “^T^”

To determine the phylogenetic relationship between these fungi and allied species from NCBI we constructed a phylogenetic tree based on Maximum Likelihood (ML) and Bayesian analysis, based on concatenated sequences of five genes included 89 taxa, comprising 4491 characters (SSU: 1047bp, LSU: 849 bp, *TEF*: 945bp, *RPB1*: 717 bp, *RPB2*: 933bp). The multi-gene phylogenetic tree consisted of four genera belonging to the family Cordycipitaceae, including *Akanthomyces*, *Beauveria*, *Cordyceps* and *Samsoniella*, with strong support (100%). Statistical support (≥75%/0.75) is shown at the nodes for ML bootstrap support/BI posterior probabilities and the strains’ numbers are noted after each species’ name (Fig. 1).

In the phylogenetic tree, *Samsoniella* species clustered in a clade easily distinguished from species of *Akanthomycessensu stricto*, *Beauveria* and *Cordyceps*. Within the *Samsoniella* clade, the majority of *Samsoniella* species grouped together, while only two strains, named as *S.lepidopterorum*, formed a separate branch with a relatively far genetic distance. Furthermore, the seven spider- pathogenic strains (RCEF 0643, RCEF 1481, RCEF 2831, RCEF 2868, RCEF 2588, RCEF 2830, RCEF 2595) and four insect- pathogenic strains (RCEF2590, RCEF 2592, RCEF 2870, RCEF 5406) in this study are located on different branches of the *Samsoniella* clade. Strains RCEF 0643 and *S.alpina* were clustered in the same branch (MLBP=98, PP=1.00). Strain RCEF 2592 and RCEF 2595 were grouped with *S.erucae* clade (MLBP=95,PP=1.00). Strain RCEF 1481 was clustered in the same clade with *S.hepiali* (MLBP=100,PP=1.00). However, another seven strains formed three independent branches. *S.fusiformispora* (RCEF 5406 and RCEF 2588) formed a monophyletic group which closely clustered with *S.hymenopterorum* and *S.farinosa* with high bootstrap values. *S.aranea* (RCEF 2831 RCEF 2868, and RCEF 2870) clustered in an independent branch, which was phylogenetically close to *S.yunnanensis* (MLBP=100,PP=1.00). *S.anhuiensis* (RCEF 2830 and RCEF 2590) formed an independent sister branch with high support(MLBP=97, PP=0.97). Five-gene phylogenetic analyses suggested that RCEF 0643, RCEF 1481, RCEF 2592, and RCEF 2595 were known species. However, the other seven strains were three new species in *Samsoniella*.

### ﻿Taxonomy

#### ﻿ Samsoniella anhuiensis

Taxon classificationFungiHypocrealesCordycipitaceae

T. Wang, Ming J. Chen & B. Huang sp. nov.

D49C7ADA-A2B5-53E6-9B40-4A243C9C7384

849801

Fig. 2

##### Etymology.

Named after the location Anhui Province where the species was originally collected.

##### Typification.

China. Anhui Province: Xuancheng City, the Jingting Mountains National Forest Park, on a spider attached to a leaf, 15 March 2006, Mingjun Chen & Xueqiu Zhao, holotype XC20060315-06. Sequences from strain RCEF2830 and RCEF2590 have been submitted to GenBank with accession numbers. RCEF2830: SSU = OM268844; LSU = OM268849; *TEF* = OM483865; *RPB1* = OM751889. RCEF2590: SSU = OR978313; LSU = OR978316; *TEF* = OR966516; *RPB1* = OR989964.

##### Description.

**Figure 2.**
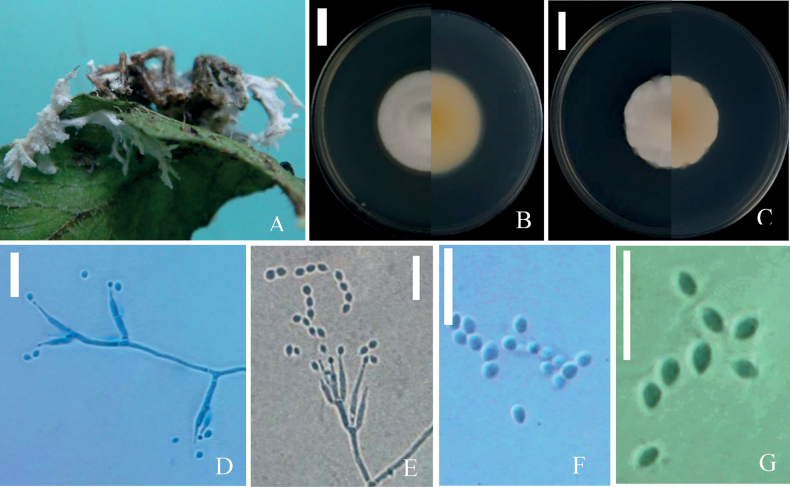
*Samsoniellaanhuiensis***A** fungus on spider **B** colony on SDAY/4 **C** colony on PDA**D, F** conidiophores structure and conidia on SDAY/4 **E, G** conidiophores structure and conidia on PDA. Scale bars: 15 mm (**B, C**); 10 μm (**D–G**).

***Sexual morph***: Undetermined. ***Asexual morph***: *Isaria*-like. Synnemata arising from the whole body of spider, white, flexuous, multiple, fleshy, up to 12 mm long, with terminal branched, white conidia produced from the branches of synnemata, powdery and floccose (Fig. 2A). Conidiophores arising from the aerial and prostrate hyphae, solitary and verticillate. Phialides in whorls of 2–5, 5.0–15.2 × 1.5–2.3 μm, smooth-walled, with basal portion swollen to ellipsoidal, tapering into a distinct neck, 1.8–5.2 × 0.8–1.2 μm. Conidia in chains, spherical to elliptical, aseptate, hyaline, 2.1–3.2 × 1.3–2.2 μm.

##### Culture characteristics.

Colonies on 1/4 SDAY, attaining a diam 38–42 mm in 14 d at 25 °C. Colonies white, with smooth and neat edge, with high mycelial density at the centrum (Fig. 2B). Reverse pale yellow to yellowish, appears flesh pink at 30 d. Hyphae smooth, septate, hyaline, 1.5–2.3 μm width. Erect conidiophores usually arising from aerial hyphae, with phialides in whorls of two to three or occasionally with solitary phialides along the hyphae. Phialides basal portion cylindrical, tapering to a distinct neck, 4.8–16.0 μm long, 1.4–2.0 μm basal width and 0.6–1.0 μm distinct neck width. Conidia in (Fig. 2D), smooth-walled, hyaline, spherical to elliptical, ovoid, occasionally pointed at both ends, 2.4–3.2 × 1.5–2.1 μm (Fig. 2F). Chlamydospores and synnemata not observed.

Colonies on PDA, 39–41mm diameter in 14 d at 25 °C, white. The central part of the colony is raised and appears light yellowish (Fig. 2C). Reverse yellowish in the center. Hyphae smooth, septate, hyaline, with septum and branches, 1.5–2.8 μm width, with phialides in whorls of two to five. Phialides basal portion cylindrical, tapering to a distinct neck, (7-)8–11.5(-13) μm long, 1.3–2.2 μm basal width and 0.5–0.8 μm distinct neck width (Fig. 2E). Conidia in chains, 1-celled, smooth-walled, hyaline, fusiform, elliptical, to obovate, 2–3(-3.5) × 1–2.5 μm (Fig. 2G).

##### Habitat.

Occurring on spider attached to the upperside of tree leaf.

##### Notes.

*Samsoniellaanhuiensis* was easily identified as belonging to *Samsoniella* based on the phylogenetic analyses (Fig. 1). Based on the combined multigene dataset, *S.anhuiensis* has an independent branch and has a close relationship with *S.tiankengensis*. However, colonies of *S.tiankengensis* exhibit a faster growth rate on PDA compared to *S.anhuiensis*, displaying white to light pink colonies with a light yellowish reverse. In contrast, colonies of *S.anhuiensis* appear light yellowish and take on a flesh-pink hue at 30 days on 1/4 SDAY, with a yellowish center in reverse. Notably, *S.anhuiensis* distinguishes itself from S.tiankengensis through the presence of larger spherical, elliptical to ovoid conidia (Table 2).

**Table 2.** Morphological comparison of three new species with other related *Samsoniella* species (Wang et al. 2022).

Species	Morphological characteristics							Reference
	Synnemata (mm)	Conidiophores (μm)	Colony growth rate (mm)(14d, 25 °C)	Phialide	Phialides size (μm)	Conidia (μm)	Hosts/substrates	
***S.anhuiensis***	white, flexuous, multiple, fleshy, up to 12, with terminal branched	-	39–41	verticillate, in whorls of 2 to 5	8.0–11.5 × 1.3–2.2, , wide (apex) 0.5–0.8, basal portion cylindrical to narrowly lageniform	Fusiform, spherical, to obovate 2.0–3.5 × 1.0–2.5	spider	this study
*S.alpina*	irregularly branched, 3–20 long, cylindrical or clavate stipes with white powdery heads	3.1–6.5 × 1.6–2.8	up to 40	verticillate on conidiophores, solitary or verticillate on hyphae	4.7–9.5 × 1.9–3.1, wide (apex) 0.5–1.1, basal portion cylindrical to narrowly lageniform	fusiform or oval 2.0–3.1 × 1.3–2.1	larvae of *Hepialusbaimaensis*	Wang et al. 2020a
***S.aranea***	Synnemata not observed	-	34.5–36	verticillate, in whorls of 2 to 4	6.9–11.2 × 1.4–1.9, wide (apex) 0.5–0.9, basal portion cylindrical to narrowly lageniform	elliptical, fusiform 1.9–3.4 × 1.2–2.4	spider	this study
*S.coleopterorum*	Synnemata not observed	-	36–40	verticillate, in whorls of 2 to 4	5.4–9.7 × 1.2–1.8, a cylindrical to ellipsoidal basal portion	fusiform, ellipsoidal or subglobose 1.7–2.5 × 1.2–1.8	Snout beetle Curculionidae	Chen et al. 2020
*S.erucae*	branched or unbranched, fleshy	-	46–48	solitary or in groups of three	6.8 -13.7 × 1.1 -1.5 with a cylindrical or ellipsoidal basal portion and tapered into a short, distinct neck	fusiform to ellipsoidal 2.3–2.9 × 1.1–1.5	caterpillar Lepidoptera	Chen et al. 2022
***S.fusiformispora***	multiple, unbranched, 2–3 long	-	36.5–39	verticillate, in whorls of 2 to 5	7.4–16.0 × 1.3–1.9, wide (apex) 0.5–1.0, basal portion cylindrical to narrowly lageniform	fusiform 1.9–3.4 × 1.2–2.4	spider	this study
*S.hepiali*	branched or unbranched, 5–41long	4.0–7.6 × 1.4–2.2	50–55	verticillate, in whorls of 2 to 5, solitary or opposite on hyphae	3.5–13.6 × 1.3–2.1, wide (apex) 0.5–1.0, basal portion cylindrical to narrowly lageniform	fusiform or oval 1.8–3.3 × 1.4–2.2	larvae of *Hepialusarmoricanus*	Wang et al. 2020a
*S.tiankengensis*	branched or unbranched, fleshy	-	53–56	solitary or in groups of four	5.4–10.4 × 1.3–2.2, cylindrical or subellipsoidal basal portion and tapered into a short, distinct neck	ellipsoidal 2.3–2.8 × 1.6–1.8	pupa of Lepidoptera	Chen et al. 2022
*S.yunnanensis*	gregarious, flexuous, fleshy, 4.0–18.0 long, with terminal branches of 3–7 × 1.0–2.0	4.2–23.5 × 1.4–2.3	48–50	verticillate, in whorls of 2 to 7, usually solitary on hyphae	4.5–11.6 × 1.2–2.4, wide (apex) 0.6–1.0, basal portion cylindrical to narrowly lageniform	fusiform or oval 2.0–3.3 × 1.1–2.2	pupa of Limacodidae	Wang et al. 2020a

#### ﻿ Samsoniella aranea

Taxon classificationFungiHypocrealesCordycipitaceae

T. Wang, Ming J. Chen & B. Huang sp. nov.

58AEB248-4ADD-5D01-A852-2E2CBBBB5703

849800

Fig. 3

##### Etymology.

Referring to its host, spider, family Araneae.

##### Typification.

China. Anhui Province: Xuancheng City, the Jingting Mountains National Forest Park, on spiders, in the litter layer, 15 March 2006 and 27 April 2006, Mingjun Chen & Xueqiu Zhao, holotype XC20060427-06, ex-holotype XC20060315-12. Sequences from strains RCEF2868, RCEF2831 and RCEF 2870 have been submitted to GenBank with accession numbers: RCEF2868: SSU = OM268846; LSU = OM268851; *TEF* = OM483867; *RPB1* = OM751883; *RPB2* = OM802501. RCEF2831: SSU = OM268845; LSU = OM268850; *TEF* = OM483866; *RPB1* = OM751882; *RPB2* = OM802500. RCEF 2870: SSU = OR978314; LSU = O978317; *TEF* = OR966517; *RPB1* = OR989965; *RPB2* = OR989966.

##### Description.

**Figure 3.**
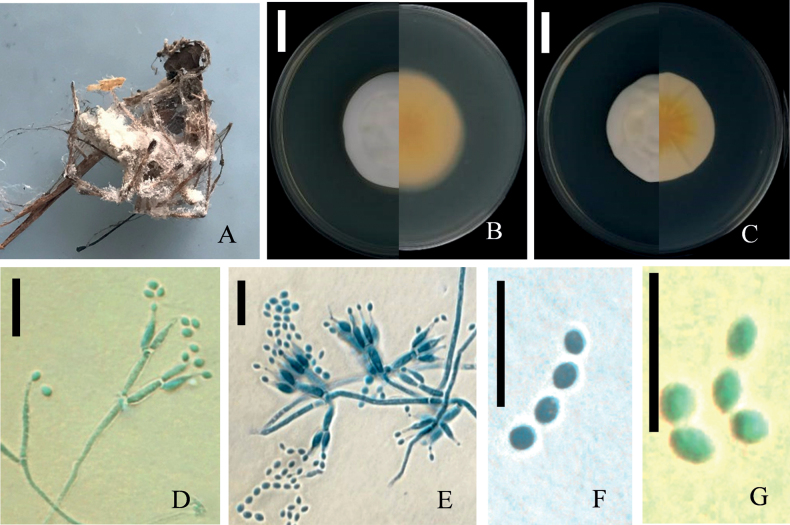
*Samsoniellaaranea***A** fungus on spider **B** colony on SDAY/4 **C** colony on PDA**D, G** conidiophores structure and conidia on SDAY/4 **E, F** conidiophores structure and conidia on PDA. Scale bars: 15 mm (**B, C**); 10 μm (**D–G**).

***Sexual morph***: Undetermined. ***Asexual morph***: *Isaria*-like. Mycellium on the spider consisting of white, smooth, branched, septate, 1.6–2.5 μm diam hyphae (Fig. 3A). Conidiophores solitary, arising from superficial hyphae, smooth, cylindrical, flexuous. Phialides verticillate, in whorl of 2–4, 5.0–12.6 × 1.2–2.3 μm, with basal portion swollen to ellipsoidal, tapering into a distinct neck, 4.0–6.0 × 0.8–1.0μm. Conidia in chains, fusiform, aseptate, hyaline, 2.1–3.6 × 1.5–2.4 μm.

##### Culture characteristics.

Colonies on 1/4 SDAY, attaining a diam of 34.5–41.0 mm in 14 d at 25 °C, floccose, colonies white to cream-yellowish, with white smooth and neat edge (Fig. 3B), reverse light yellowish, sporulating abundantly. Hyphae smooth-walled, branched, hyaline, septate, 1.5–2.3 μm wide. Conidiophores smooth-walled, cylindrical, verticillate, 4.8–16.0 × 1.4–2.0 μm. Phialides in whorls of two to four, usually solitary on hyphae, basal portion cylindrical, tapering to a distinct neck; 5.1–16.9 μm long, 1.3–2.1 μm wide at the base, and 0.5–1.0 μm wide at the apex (Fig. 3D). Conidia in chains, smooth-walled, hyaline, elliptical, occasionally fusiform, 1.9–3.5 × 1.4–2.6 μm (Fig. 3G). Chlamydospores and synnemata not observed.

Colonies on PDA, attaining a diam of 34.5–36 mm in 14 d at 25 °C, floccose, colonies white to cream-yellowish, with a white smooth and neat edge, forming radial folds from the center outwards (Fig. 3C). Reverse yolk yellowish, sporulating abundantly. Hyphae smooth walled, branched, hyaline, septate, 1.5–2.6 μm wide. Conidiophores smooth – walled, cylindrical, verticillate. Phialides in whorls of two to four, usually solitary on hyphae, basal portion cylindrical, tapering to a distinct neck; 6.9–11.2 μm long, 1.4–1.9 μm wide at the base, and 0.5–0.9μm wide at the apex (Fig. 3E). Conidia 1-celled, in chains, smooth-walled, hyaline, elliptical, occasionally fusiform, 1.9–3.4 × 1.2–2.4 μm (Fig. 3F).

##### Habitat.

Occurring on spiders in the litter layer.

##### Notes.

*Samsoniellaaranea* was readily classified within the genus *Samsoniella* through phylogenetic analyses (Fig. 1). Analysis of the combined multigene dataset unveiled that *S.aranea* forms an independent branch and shares a close relationship with *S.yunnanensis*. However, notable distinctions were observed between the two species. Unlike *S.yunnanensis*, where synnemata arise from insect cocoons, synnemata of *S.aranea* were not observed. Additionally, distinct growth characteristics were noted, with colonies of *S.yunnanensis* exhibiting a faster growth rate on PDA compared to *S.aranea*. Morphological differences were evident in the colonies on PDA, with *S.aranea* colonies being floccose, white to cream-yellowish, and having a yolk-yellowish reverse. On the other hand, colonies of *S.yunnanensis* were described as loose and hairy, appearing white with a reddish-brown reverse.

#### ﻿ Samsoniella fusiformispora

Taxon classificationFungiHypocrealesCordycipitaceae

T. Wang, Ming J. Chen & B. Huang sp. nov.

3056CCD8-C104-5679-A5D1-706E27536B4B

849799

Fig. 4

##### Etymology.

Referring to the typical fusiform conidia.

##### Typification.

China. Gansu Province: Tianshui City, Maiji National Forest Park, on a spider, underside of tree leaf, 22 September 2010, Wang Liming, holotype MJS20100922-21. Sequences from strain RCEF5406 and RCEF2588 submitted to GenBank with accession numbers. RCEF5406: SSU = OM268843; LSU = OM268848; *TEF* = OM483864; *RPB1* = OM751890. RCEF2588: SSU = OR978312; LSU = OR978315; *TEF* = OR966515.

##### Description.

**Figure 4.**
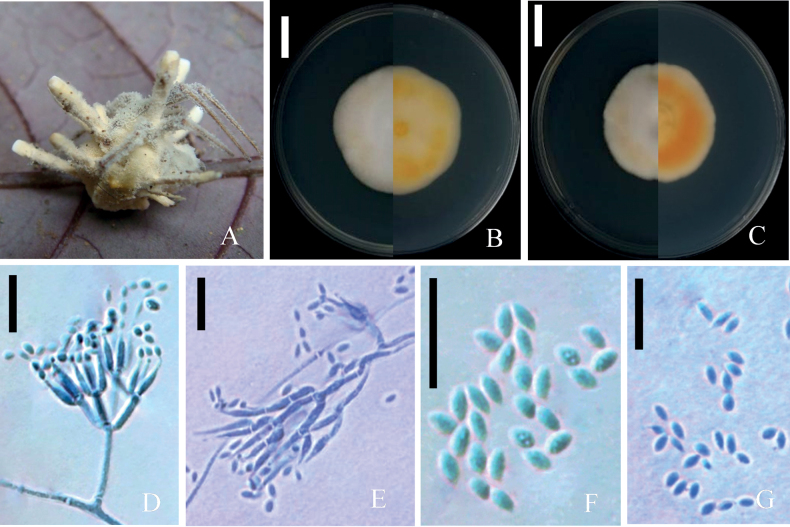
*Samsoniellafusiformispora***A** fungus on spider **B** colony on SDAY/4 **C** colony on PDA**D, F** conidiophores structure and conidia on SDAY/4 **E, G** conidiophores structure and conidia on PDA. Scale bars: 15 mm (**B, C**); 10 μm (**D–G**).

***Sexual morph***: Undetermined. ***Asexual morph***: *Isaria*-like. Synnemata multiple, unbranched, arising from the whole body of spider, 3–6 mm long, Stipes cylindrical or clavate, 0.5–1.0 mm wide, pale yellowish, white conidia produced from the synnemaya and hyphal layer (Fig. 4A). Phialides verticillate, in whorl of 2–5, 5.0–12.0 × 1.9–2.8 μm, with basal portion swollen to ellipsoidal, tapering into a distinct neck, 2.3 -3.8 × 0.5–1.2 μm. Conidia in chains, fusiform, aseptate, hyaline, 2.1–3.5 × 1.6–2.2 μm.

##### Culture characteristics.

Colonies on 1/4 SDAY fast-growing, 39.5–44 mm diameter in 14 d at 25 °C, colonies white edge to yellowish center, cottony (Fig. 4B), reverse yellow to orange-yellow, hyphae smooth – walled, branched, hyaline, septate, 1.7–2.6 μm wide. Conidiophores smooth-walled, cylindrical, verticillate. Phialides in whorls of three to five, usually solitary on hyphae, basal portion cylindrical, tapering to a distinct neck; 7.6–15 μm long, 1.9–2.6 μm wide at the base, and 0.7–1.2 μm wide at the apex (Fig. 4D). Conidia in chains, smooth-walled, hyaline, fusiform, 2.1–3.6(-4.4) × 1.8–2.2 μm (Fig. 4F). Chlamydospores and synnemata not observed. Size and shape of phialides and conidia similar in culture. Sexual state not observed.

Colonies on PDA, attaining a diam of 36.5–39 mm in 14 d at 25 °C, floccose, colonies white to yellowish, with high mycelial density at the centrum (Fig. 4C). Reverse pale yellowish edge to orang center. Hyphae smooth- walled, branched, hyaline, septate, 1.5–2.5 μm wide. Conidiophores smooth – walled, cylindrical, verticillate. Phialides in whorls of two to five, usually solitary on hyphae, basal portion cylindrical, tapering to a distinct neck; 7.4–16(–26) μm long, 1.3–1.9(–2.4) μm wide at the base, and 0.5–1.0 μm wide at the apex (Fig. 4E). Conidia 1-celled, in chains, smooth-walled, hyaline, fusiform, 1.9–3.4 × 1.2–2.4 μm (Fig. 4G).

##### Habitat.

Occurring on spider attached to the underside of tree leaf.

##### Notes.

*Samsoniellafusiformispora* was unequivocally identified as a member of the Samsoniella genus through phylogenetic analyses (Fig. 1) and was found to share a close relationship with *S.coleopterorum*. However, upon further investigation and comparison of the morphological characteristics of the three new species with other related *Samsoniella* species (Table 2), distinct differences emerged. Colonies of *S.fusiformispora* were noted to be white to yellowish, with a pale yellowish edge transitioning to an orange center in reverse. In contrast, colonies of *S.coleopterorum* were observed to be white, with a yellowish reverse.

## ﻿Discussion

The typical characteristics of *Samsoniella* were oval to fusiform conidia, bright red-orange stromata of the sexual morphs and synnemata of the asexual morphs (Chen et al. 2020). In this study, we present a phylogenetic investigation of cordycipitaceous *Isaria*-like fungi pathogenic on spiders. Combined with microscopic characteristics and phylogenetic analysis based on multi-locus sequence data, *S.fusiformispora*, *S.aranea* and *S.anhuiensis* were described and illustrated as new species in *Samsoniella*. It was found that the hosts of most reported *Samsoniella* species are Lepidoptera larvae or pupae, while the host of *S.coleopterorum* is a snout beetle (Curculionidae), and the host of *S.hymenopterorum* is a bee (Mongkolsamrit et al. 2018; Chen et al. 2020; Wang et al. 2020a). However, it should be noted that Wang et al. (2020a) described the host of *S.hymenopterorum* as being “Bee, family Vespidae”. The family Vespidae are wasps, not bees. Our study has expanded the hosts of *Samsoniella* from Insecta to Arachnida.

Generally, the phialides of *S.fusiformispora* were longer and thinner than those of the closely-related *S.coleopterorum* while they also had bigger typical fusiform conidia with greater length to width ratio. In the ML and BI phylogenetic trees, *S.aranea* was inferred as a phylogenetic sister of *S.yunnanensis* with strong support (93%/1.00) and distinct from other related species in *Samsoniella*. The synnemata of *S.aranea* was not observed, but *S.yunnanensis* has gregarious, flexuous and fleshy synnemata arising from the limacodid cocoons (Wang et al. 2020a). Furthermore *S.yunnanensis* has smaller fusiform to oval conidia than *S.aranea* and the colonies on PDA grow faster than *S.aranea*. Similarly, *S.anhuiensis* was easily separated by the phylogenetic analyses with independent branches in the phylogenetic tree.

Kepler et al. (2017) found that sequences of *Isaria* sp. spat 09-050 and *Isaria* sp. spat 09-051 were firstly obtained, and two strains were clustered as the phylogenetic sister of *Isaria* spp. with 100 bootstrap proportion in the weighted parsimony (WP) analytic tree based on five genes (SSU, LSU, TEF, *RPB1* and *RPB2*), which was classified as *Akanthomyces* group. Then Wang et al. (2020a) constructed the multigene phylogenetic tree studied the new taxa of the family Cordycipitaceae and the new systematic position of the Chinese cordycipitoid fungus *Paecilomyceshepiali*. In this multigene phylogenetic tree, *Isaria* sp. spat 09-050 and *Isaria* sp. spat 09-051 were clustered in genus *Samsoniella* as sister group of *S.vallis* but in two independent branches. In this study, we obtained the same results. We convinced that *Isaria* sp. spat 09-050 and *Isaria* sp. spat 09-051 is an unpublished new species of the *Samsoniella*, should be revised to *Samsoniella* sp. spat 09-050 and *Samsoniella* sp. spat 09-051.

In this study, based on morphological characteristics and five loci phylogenetic analysis, *S.anhuiensis*, *S.aranea* and *S.fusiformispora* were separated from other *Samsoniella* species, which are described here as new species. The strain RCEF0643 was identified as *S.alpina*, the strain RCEF1481 was named as *S.hepiali*, and the strains RCEF2592 and RCEF 2590 was identified as *S.erucae*. Furthermore, our study significantly broadens the host range of *Samsoniella* from Insecta to Arachnida, marking a noteworthy expansion in understanding the ecological associations of these fungi. Additionally, the identification of both mononematous and synnematous conidiophores in our study not only expands the knowledge of *Samsoniella* species but also provides a basis for future research by comparing the ecological significance between these conidiophore types.

## Supplementary Material

XML Treatment for Samsoniella anhuiensis

XML Treatment for Samsoniella aranea

XML Treatment for Samsoniella fusiformispora

## ﻿Additional information

### Author ORCIDs

Ting Wang https://orcid.org/0000-0002-9296-7280

Jun Li https://orcid.org/0009-0009-3183-2604

Xiaoyun Chang https://orcid.org/0000-0002-0093-9582

Zengzhi Li https://orcid.org/0000-0002-9606-5030

Nigel L. Hywel-Jones https://orcid.org/0009-0004-8219-3682

Bo Huang https://orcid.org/0000-0001-6032-7396

Mingjun Chen https://orcid.org/0000-0002-1439-7796

### Data availability

All of the data that support the findings of this study are available in the main text.

## References

[B1] Bainer G (1907) Mycotheque del’ École de Pharmacie XI. *Paecilomyces*, genre noveau de Mucédinées. Bulletin de la Société Mycologique de France 23: 26–27.

[B2] Bischoff JF Rehner SA Humber RA (2009) A multilocus phylogeny of the *Metarhiziumanisopliae* lineage. Mycologia 101(4): 512–530. 10.3852/07-202 19623931

[B3] Castlebury LA Rossman AY Sung GH Hyten AS Spatafora JW (2004) Multigene phylogeny reveals new lineage for *Stachybotryschartarum*, the indoor air fungus. Mycological Research 108(8): 864–872. 10.1017/S0953756204000607 15449591

[B4] Chen WH Han YF Liang JD Tian WY Liang ZQ (2020) Morphological and phylogenetic characterisations reveal three new species of *Samsoniella* (Cordycipitaceae, Hypocreales) from Guizhou, China. MycoKeys 2020(74): 1–15. 10.3897/mycokeys.74.56655 PMC7588496 33149720

[B5] Chen WH Liang JD Ren XX Zhan JH Han YF Liang ZQ (2021) Cryptic diversity of *Isaria*-like species in Guizhou, China. Life 11(10): e1093. 10.3390/life11101093 PMC8539930 34685462

[B6] Chen WH Liang JD Ren XX Zhao JH Han YF Liang ZQ (2022) Species Diversity of *Cordyceps*-Like Fungi in the Tiankeng Karst Region of China. Microbiology Spectrum 10(5): e0197522. 10.1128/spectrum.01975-22 PMC9603550 36094103

[B7] Chen WH Liang JD Ren XX Zhao JH Han YF (2023) Two new species of *Samsoniella* (Cordycipitaceae, Hypocreales) from the Mayao River Valley, Guizhou, China. MycoKeys 99: 209–226. 10.3897/mycokeys.99.109961 37744955 PMC10517413

[B8] Crous PW Osieck ER Shivas RG Tan YP Bishop-Hurley SL Esteve-Raventós F Larsson E Luangsa-ard JJ Pancorbo F Balashov S Baseia IG Boekhout T Chandranayaka S Cowan DA Cruz RHSF Czachura P De la Peña-Lastra S Dovana F Drury B Fell J Flakus A Fotedar R Jurjević Ž Kolecka A Mack J Maggs-Kölling G Mahadevakumar S Mateos A Mongkolsamrit S Noisripoom W Plaza M Overy DP Piątek M Sandoval Denis M Vauras J Wingfield MJ Abell SE Ahmadpour A Akulov A Alavi F Alavi Z Altes A Alvarado P Anand G Ashtekar N Assyov B Banc-Prandi G Barbosa KD Barreto GG Bellanger JM Bezerra JL Bhat DJ Bilanski P Bose T Bozok F Chaves J Costa-Rezende DH Danteswari C Darmostuk V Delgado G Denman S Eichmeier A Etayo J Eyssartier G Faulwetter S Ganga KGG Ghosta Y Goh J Góis JS Gramaje D Granit L Groenewald M Gulden G Gusmão LFP Hammerbacher A Heidarian Z Hywel-Jones N Jankowiak R Kaliyaperumal M Kaygusuz O Kezo K Khonsanit A Kumar S Kuo CH Læssøe T Latha KPD Loizides M Luo SM Maciá-Vicente JG Manimohan P Marbach PAS Marinho P Marney TS Marques G Martín MP Mille AN Mondello F Moreno G Mufeeda KT Mun HY Nau T Nkomo T Okrasińska A Oliveira JPAF Oliveira RL Ortiz DA Pawłowska J Pérez-De-Gregorio MÀ Podile AR Portugal A Privitera N Rajeshkumar KC Rauf I Rian B Rigueiro-Rodríguez A Rivas-Torres GF Rodriguez-Flakus P Romero-Gordillo M Saar I Saba M Santos CD Sarma PVSRN Siquier JL Sleiman S Spetik M Sridhar KR Stryjak-Bogacka M Szczepańska K Taşkın H Tennakoon DS Thanakitpipattana D Trovao J Türkekul İ van Iperen AL van’t Hof P Vasquez G Visagie CM Wingfield BD Wong PTW Yang WX Yarar M Yarden O Yilmaz N Zhang N Zhu YN Groenewald JZ (2023) Fungal Planet description sheets: 1478–1549. Persoonia 50(1): 158–310. 10.3767/persoonia.2023.50.05 PMC10983837 38567263

[B9] Darriba D Taboada GL Doallo R Posada D (2012) jModelTest 2: More models, new heuristics and parallel computing. Nature Methods 9(8): 772–772. 10.1038/nmeth.2109 PMC4594756 22847109

[B10] Gams W Hodge KT Samson RA Korf RP Seifert KA (2005) Proposal to conserve the name *Isaria* (anamorphic fungi) with a conserved type. Taxon 54(2): e537. 10.2307/25065390

[B11] Hodge KT Gams W Samson RA Korf RP Seifert KA (2005) Lectotypification and status of *Isaria* Pers. Taxon 54(2): 485–489. 10.2307/25065379

[B12] Hopple JS (1994) Phylogenetic Investigations in the Genus Coprinus Based on Morphological and Molecular Characters. Ph.D. Dissertation, Duke University, Durham, USA.

[B13] Katoh K Standley DM (2013) MAFFT multiple sequence alignment software version 7: Improvements in performance and usability. Molecular Biology and Evolution 30(4): 772–780. 10.1093/molbev/mst010 23329690 PMC3603318

[B14] Kepler RM Luangsa-ard JJ Hywel-Jones NL Quandt CA Sung GH Rehner SA Aime MC Henkel TW Sanjuan T Zare R Chen MJ Li ZZ Rossman AY Spatafora JW Shrestha B (2017) A phylogenetically -based nomenclature for Cordycipitaceae (Hypocreales). IMA Fungus 8(2): 335–353. 10.5598/imafungus.2017.08.02.08 29242779 PMC5729716

[B15] Letunic I Bork P (2016) Interactive tree of life (iTOL) v3: An online tool for the display and annotation of phylogenetic and other trees. Nucleic Acids Research 44(W1): W242–W245. 10.1093/nar/gkw290 PMC4987883 27095192

[B16] Liu YJ Whelen S Hall BD (1999) Phylogenetic relationships among ascomycetes: Evidence from an RNA polymerse II subunit. Molecular Biology and Evolution 16(12): 1799–1808. 10.1093/oxfordjournals.molbev.a026092 10605121

[B17] Liu ZY Liang ZQ Whalley AJS Yao YJ Liu AY (2001) *Cordycepsbrittlebankisoides*, a new pathogen of grubs and its anamorph, Metarhiziumanisopliaevar.majus. Journal of Invertebrate Pathology 78(3): 178–182. 10.1006/jipa.2001.5039 11812122

[B18] Luangsa-ard JJ Hywel-Jones NL Samson RA (2004) The order level polyphyletic nature of *Paecilomyces* sensu lato as revealed through 18S-generated rRNA phylogeny. Mycologia 96: 773–780. 10.1080/15572536.2005.11832925 21148898

[B19] Luangsa-ard JJ Hywel-Jones NL Manoch L Samson RA (2005) On the relationships of Paecilomycessect.Isarioidea species. Mycological Research 109(5): 581–589. 10.1017/S0953756205002741 16018312

[B20] Maharachchikumbura SSN Hyde KD Jones EBG McKenzie EHC Huang SK Abdel-Wahab MA Daranagama DA Dayarathne M D’souza MJ Goonasekara ID Hongsanan S Jayawardena RS Kirk PM Konta S Liu JK Liu ZY Norphanphoun C Pang KL Perera RH Senanayake IC Shang QJ Shenoy BD Xiao YP Bahkali AH Kang JC Somrothipol S Suetrong S Wen TC Xu JC (2015) Towards a natural classification and backbone tree for Sordariomycetes. Fungal Diversity 72(1): 199–301. 10.1007/s13225-015-0331-z

[B21] Mongkolsamrit S Noisripoom W Thanakitpipattana D Wutikhun T Spatafora JW Luangsa-ard JJ (2018) Disentangling cryptic species with *Isaria*-like morphs in Cordycipitaceae. Mycologia 110(1): 230–257. 10.1080/00275514.2018.1446651 29863995

[B22] Persoon CH (1794) Dispositio methodica fungorum. Neues Magazin für die Botanik 1: 81–128.

[B23] Rehner SA Buckley E (2005) A *Beauveria* phylogeny inferred from nuclear ITS and EF1-α sequences: Evidence for cryptic diversification and links to *Cordyceps* teleomorphs. Mycologia 97(1): 84–98. 10.3852/mycologia.97.1.84 16389960

[B24] Ronquist F Teslenko M van der Mark P Ayres DL Darling A Höhna S Larget B Liu L Suchard MA Huelsenbeck JP (2012) MrBayes 3.2: Efficient Bayesian phylogenetic inference and model choice across a large model space. Systematic Biology 61(3): 539–542. 10.1093/sysbio/sys029 22357727 PMC3329765

[B25] Samson RA (1974) *Paecilomyces* and some allied hyphomycetes. Studies in Mycology 6: 1–119.

[B26] Smith FB (1975) Naturalist’s Color Guide. America Museum Natural History, New York, 22 pp.

[B27] Stamatakis A (2014) RAxML version 8: A tool for phylogenetic analysis and post-analysis of large phylogenies. Bioinformatics 30(9): 1312–1313. 10.1093/bioinformatics/btu033 24451623 PMC3998144

[B28] Sung GH Hywel-Jones NL Sung JM Luangsa-ard JJ Shrestha B Spatafora JW (2007) Phylogenetic classification of *Cordyceps* and the clavicipitaceous fungi. Studies in Mycology 57(1): 5–59. 10.3114/sim.2007.57.01 18490993 PMC2104736

[B29] Vilgalys R Hester M (1990) Rapid genetic identification and mapping of enzymatically amplified ribosomal DNA from several *Cryptococcus* species. Journal of Bacteriology 172(8): 4238–4246. 10.1128/jb.172.8.4238-4246.1990 2376561 PMC213247

[B30] Wang YB Wang Y Fan Q Duan DE Zhang GD Dai RQ Dai YD Zeng WB Chen ZH Li DD Tang DX Xu ZH Sun T Nguyen TT Tran NL Dao VM Zhang CM Huang LD Liu YJ Zhang XM Yang DR Sanjuan T Liu XZ Yang ZL Yu H (2020a) Multigene phylogeny of the family Cordycipitaceae (Hypocreales): New taxa and the new systematic position of the Chinese cordycipitoid fungus *Paecilomyceshepiali*. Fungal Diversity 103(1): 1–46. 10.1007/s13225-020-00457-3

[B31] Wang Y Tang DX Duan DE Wang YB Yu H (2020b) Morphology, molecular characterization, and virulence of *Beauveriapseudobassiana* isolated from different hosts. Journal of Invertebrate Pathology 172: e107333. 10.1016/j.jip.2020.107333 32001215

[B32] Wang Z Wang Y Dong Q Fan Q Dao VM Yu H (2022) Morphological and phylogenetic characterization reveals five new species of *Samsoniella* (Cordycipitaceae, Hypocreales). Journal of Fungi 8(7): e747. 10.3390/jof8070747 PMC9321185 35887502

[B33] Wang Y Wang ZQ Thanarut C Dao VM Wang YB Hong Y (2023) Phylogeny and species delimitations in the economically, medically, and ecologically important genus *Samsoniella* (Cordycipitaceae, Hypocreales). MycoKeys 99: 227–250. 10.3897/mycokeys.99.106474 37828936 PMC10565569

[B34] White TJ Bruns T Lee S Taylor J (1990) Amplification and direct sequencing of fungal ribosomal RNA genes for phylogenetics. PCR protocols: a guide to methods and applications 18(1): 315–322. 10.1016/B978-0-12-372180-8.50042-1

